# Current News

**Published:** 1902-08

**Authors:** 


### Current News.

### HARVARD DENTAL ALUMNI ASSOCIATION.

AT the thirty-first annual meeting held June 23, 1902, the following officers were elected for the ensuing year:

President, Luther D. Shepard, '79, Boston, Mass.; Vice-President, Charles E. Perkins, '90, Brockton, Mass.; Secretary, Waldo E. Boardman, '86, Boston, Mass.; Treasurer, E. Proctor Holmes, '88, Boston, Mass.

*Executive Committee.*—Waldo E. Boardman, '86, Boston, Mass., Chairman *ex-officio;* William P. Cooke, '81, Boston, Mass.; Ned A. Stanley, '84, Boston, Mass.

The Council is composed of the officers of the Association.

WALDO E. BOARDMAN,
*Secretary.*

---

### COLORADO STATE DENTAL ASSOCIATION.

AT the sixteenth annual meeting of the Colorado State Dental Association, held in the Alta Vista Hotel, Colorado Springs, June 17, 18, and 19, 1902, the following officers were elected for the ensuing year: President, H. B. Hayden, Colorado Springs; Vice-President, E. W. Varley, Pueblo; Secretary, W. A. Brierley,

Denver; Treasurer, Wm. Smedley, Denver. The candidates elected for appointment by the governor on the State Board of Dental Examiners were W. H. Hall, Denver; H. F. Hoffman, Denver; M. H. Smith, Denver; Theodore Ashley, Cannon City; Geo. R. Warner, Grand Junction.

The next meeting will be held in Pueblo, June 16, 17, and 18, 1903.

---

### TEXAS STATE DENTAL ASSOCIATION.

The Texas State Dental Association met in the city of Waco, May 13, 14, and 15, 1902, and held a very fine meeting.

The attendance was large, and the papers and clinics were of a high order.

The next meeting will be held in the city of Houston, May, 1903.

The following officers were elected: President, Dr. J. G. Fife, Dallas; First Vice-President, Dr. Thos. P. Williams, Houston; Second Vice-President, Dr. R. D. Griffis, Paris; Secretary and Treasurer, Dr. Bush Jones, Dallas; Curator of Museum, Dr. A. F. Sontag, Waco.

*Executive Committee.*—Dr. W. R. Rathbone, Chairman, Cuero; Dr. A. J. Beville, Waco; Dr. C. O. Webb, Crockett.

---

### G. V. BLACK DENTAL CLUB.

At the annual meeting of the G. V. Black Dental Club, of St. Paul, held at Austin, Minn., June 20, 1902, the following officers were elected for the ensuing year:

President, Dr. S. Bond, Anoka, Minn.; Vice-President, Dr. J. M. Walls, St. Paul, Minn.; Secretary and Treasurer, Dr. Robert B. Wilson, St. Paul, Minn.

*Board of Censors.*—Dr. A. C. Searl, Owatonna; Dr. G. F. Andrews, St. Paul; Dr. A. M. Lewis, Austin.

Robert B. Wilson,
*Secretary and Treasurer.*

